# Characteristics and clinical course of thyroid abnormalities arisen in long term survivors of childhood cancer

**DOI:** 10.1186/s12887-023-03900-x

**Published:** 2023-03-18

**Authors:** Hye Young Jin, Jun Ah. Lee, Meerim Park, Dong-Eun Lee, Hyeon Jin Park

**Affiliations:** 1 Department of Pediatrics, Center for Pediatric Cancer, National Cancer Center, 323 Ilsan-ro, Ilsandong-gu, Goyang-si, Gyeonggi-do 10408 Republic of Korea; 2 Biostatistics Collaboration Team, Research Institute, National Cancer Center, Goyang-si, Gyeonggi-do Republic of Korea

**Keywords:** Childhood cancer survivors, Irradiation, Stem cell transplantation, Thyroid abnormalities

## Abstract

**Background:**

Thyroid abnormality is a common late effect seen in childhood cancer survivors (CCSs). We analyzed the prevalence and risk factors of thyroid abnormalities based on diagnoses and treatment modalities in CCSs.

**Methods:**

The medical records of 257 CCSs who were diagnosed with cancer less than 20 year of age were retrospectively reviewed. The median age was 11.8 years (0.1–19.8). The median follow-up period after completion of therapy was 9.6 years (5.0–19.5).

**Results:**

Of 257 subjects, thyroid abnormalities were identified in 107 (41.6%). Sixty-five out of 257 (25.3%) had subclinical hypothyroidism, and 16 (6.2%) developed central hypothyroidism. Five CCSs (1.9%) had primary overt hypothyroidism. Five (1.9%) and 6 (2.3%) CCSs were diagnosed with autoimmune thyroiditis and thyroid cancer, respectively. Among the different diagnostic groups, thyroid abnormalities were frequent in the brain tumor or Hodgkin disease or nasopharyngeal cancer groups. CCSs who received irradiation directly or near hypothalamus-pituitary-thyroid (HPT) axis had more thyroid abnormalities compared to the rest CCSs (*P* < 0.0001). CCSs who were treated with SCT had an increased prevalence of thyroid abnormalities (60.5%) compared to the other CCSs (37.9%) (*P* = 0.0069). Forty-five (42%) of 107 subjects with thyroid abnormalities had normalized thyroid hormone levels at the last follow-up. Irradiation directly or near HPT axis were thought to be a predicting factor of persistent subclinical hypothyroidism.

**Conclusions:**

Subclinical hypothyroidism was common in CCSs. CCSs with irradiation directly or near HPT axis were at risk for persistent thyroid dysfunction.

**Supplementary Information:**

The online version contains supplementary material available at 10.1186/s12887-023-03900-x.

## Introduction

Thyroid disorders are the most frequent endocrine complications in childhood cancer survivors (CCSs) [1]. The cumulative incidence of thyroid disorders is increasing in CCSs, and thyroid abnormalities were reported in up to 66% of CCSs in previous studies [2, 3]. The prevalence of primary hypothyroidism in the overall population of survivors ranged from 13.8 to 20.8% [2, 4–7]. Central hypothyroidism is commonly identified, especially among brain tumor survivors. Few studies have reported on thyroid disorders in long-term CCSs in Korea. A previous study showed that subclinical hypothyroidism was the most common (24.6%), and brain or nasopharyngeal cancer, lymphoma, and head radiation were risk factors for persistent hypothyroidism [8]. Three-quarters of the medulloblastoma and peripheral neuroectodermal tumor (PNET) survivors showed thyroid dysfunction in, and over half (56%) had permanent thyroid dysfunction [9]. The frequency of thyroid dysfunction may differ depending upon the diagnosis and treatment modalities [10]. Radiotherapy has been reported as a risk factor for hypothyroidism and thyroid nodules [3]. Chemotherapeutic agents such as busulfan and cyclophosphamide were associated with transient and often, mild forms of hypothyroidism [11]. Tyrosine kinase inhibitors (TKIs) and immune check-point inhibitors used as targeted therapy in some cancers are known to have adverse effects related to the thyroid [12]. The risk of thyroid cancer was also reported to be increased after radiotherapy for childhood cancer [13]. This study is aimed to comprehensively investigate the characteristics and clinical course of thyroid abnormalities based on diagnoses and treatment modalities in CCSs.

## Methods/design

### Subjects

Medical records were retrospectively reviewed to obtain the demographic and medical characteristics of CCSs. We reviewed the diagnoses and treatment modalities including chemotherapy, radiotherapy, and hematopoietic stem cell transplantation (SCT). The patients who visited National Cancer Center between January 2013 and August 2021 were included in the study. They were diagnosed with cancer at younger than 20 year of age. CCSs who were follow-up for at least 5 years after completion of cancer therapy were enrolled. Patients who received any cancer therapy including chemotherapy, radiotherapy, or targeted therapy such as TKIs and immune check-point inhibitors within 5 years prior to the last follow-up were excluded. The patients with residual tumor after completion of cancer therapy were also ruled out other than 3 patients with inoperable but stationary CNS tumors (2 optic nerve glioma, 1 pilocytic astrocytoma).

The subjects were categorized into 6 subgroups including leukemia or myelodysplastic syndrome (MDS), Hodgkin lymphoma (HD), non-Hodgkin lymphoma (NHL), brain tumor, nasopharyngeal cancer, and other tumors based on the diagnosis. Other tumors were sarcomas, Wilms tumor, neuroblastoma, Langerhans cell histiocytosis, and ovarian tumors.

The subjects were further classified into 3 subgroups according to the type of cancer therapy, which included chemotherapy without irradiation (chemotherapy only), irradiation group 1 and irradiation group 2. The irradiation fields in the irradiation group 1 were direct to thyroid gland or near hypothalamus-pituitary-thyroid (HPT) axis, which were as follows: head and neck, craniospinal irradiation (CSI), total body irradiation (TBI), cervical spine, nasopharynx, and mediastinum. The remaining irradiation fields (abdomen, pelvis, and limbs) were regarded as irradiation group 2. The two irradiation groups received combinations of chemotherapy.

CSI was applied for some brain tumors such as medulloblastomas, germ cell tumors, atypical teratoid/rhabdoid tumors (ATRTs), and peripheral neuroectodermal tumors (PNETs). The CSI dosage ranged from 1980 to 3960 cGy. The boosting dose varied from 900 to 3240 cGy, and thus, a maximal dose of 3960–5580 cGy at each site. The partial cranial irradiation dose ranged from 1980 to 7600 cGy, and the boosting dose varied from 540 to 1980 cGy. The maximal dose at a specific site ranged from 30 to 76 Gy. Prophylactic cranial irradiation was performed in leukemia patients (*n* = 5), and the dose of irradiation was 12 or 24 Gy. TBI was used in conditioning for SCT, and the dosage was 10 or 12 Gy. The irradiation dose for the head and neck, nasopharynx, and mediastinum ranged from 30 to 71 Gy.

To analyze the additional risk of SCT, the patients were categorized into 4 groups, which were SCT accompanied irradiation near HPT axis or TBI (SCT & irradiation group 1), SCT without irradiation, irradiation group 1 without SCT (no SCT & irradiation group 1), and the remained CCSs (chemo only or irradiation group 2).

### Thyroid function tests and thyroid dysfunction

Free thyroxine (fT4) and thyroid-stimulating hormone (TSH) were measured at the time of diagnosis, at the end of treatment, and usually, annually thereafter. Neck palpation was also performed annually. Thyroid ultrasound was conducted when abnormal finding was identified in annual surveillance of thyroid or in case of radiotherapy directly or near HPT axis. FT4 and TSH were measured using a chemiluminescent immunoassay (ADVIA Centaur XP, Siemens, Erlangen, Germany). The reference values were 80–200 ng/dL for fT4, and 0.3–4.5 mU/L for TSH. CCSs with results outside the reference value limits were defined as having thyroid dysfunction and followed-up with thyroid hormone testing. Thyroid dysfunction was categorized into subgroups of primary overt hypothyroidism, subclinical hypothyroidism, and central hypothyroidism. Low fT4 and increased TSH were defined as primary overt hypothyroidism, which was divided into two subgroups according to a TSH cutoff level of 10 mU/L. Low fT4 and normal TSHs level were defined as central hypothyroidism. Normal fT4 and elevated TSH were defined as subclinical hypothyroidism, which was also divided into two subgroups based on a TSH level of 10 mU/L.

Thyroxine medication was usually prescribed for fT4 levels below 80 ng/dL or TSH levels exceeding ≥10 mU/L with consideration of the clinical symptoms and patient’s age. Thyroxine medication or abnormal thyroid hormone levels (out of the reference range) at the last follow-up were designated as persistent thyroid dysfunction. If thyroid hormone levels normalized without medication at the last follow-up, the condition was regarded as transient dysfunction. Autoimmune thyroiditis was defined when thyroid dysfunction was present with positive antithyroid peroxidase antibodies (TPOAbs) or anti-thyroglobulin antibodies (TgAbs), which were measured by a radioimmunoassay method (DIAsource Immuno Assay S.A., Louvain-la-Neuve, Belgium). TPOAbs and TgAbs over 60 U/mL were regarded as positive. Laboratory tests of TPOAbs and TgAbs were conducted when abnormal thyroid function tests were detected during follow-up period. The thyroid cancer group included CCSs who underwent total or partial thyroidectomies due to malignant pathologic findings after fine-needle aspiration biopsy regardless of the thyroid hormone levels. Thyroid dysfunction, autoimmune thyroiditis, and thyroid cancer were considered thyroid abnormalities. Thyroid nodules were not included as thyroid abnormalities in this study because ultrasound was performed in only half CCSs.

### Statistical analysis

All statistical analyses were performed using R foundation for Statistical Computing version 4.1.2 (R Core Team (2021). R: A language and environment for statistical computing, Vienna, Austria). The Chi-squared test and Fisher’s exact test were used to compare the prevalence of thyroid dysfunction according to the diagnosis and treatment modalities. Continuous variables such as age at diagnosis were assessed using the independent t-test. The time elapsed after the initial cancer diagnosis and cumulative event rates (thyroid abnormalities) were estimated using Kaplan-Meier analysis, and global tests were performed using the log-rank test. In addition, The Bonferroni method was applied to adjust for multiple comparisons of the log-rank test. A *P*-value of < 0.05 was considered statistically significant.

## Results

### Baseline characteristics of subjects

Their baseline characteristics of the CCSs are described in Table 1. The total number of subjects was 257 (male: 147, female: 110), and the mean age at diagnosis was 10.7 ± 5.3 years. The median age was 11.8 years (0.1–19.8). The median duration from initial diagnosis of cancer to the end of treatment was 0.8 years (0.3–15.1). The median follow-up period after completion of therapy was 9.6 years (5.0–19.5), and the median follow-up time from diagnosis was 11.2 years (5.4–29.5).

**Table 1:** Baseline characteristics and prevalence of thyroid abnormalities

		Total	Thyroid abnormalities		*P*-value
			No	Yes	
		(*N* = 257)	(*N* = 150)	(*N* = 107)	
Diagnosis	Leukemia/MDS	45 (17.5%)	30 (20.0%)	15 (14.0%)	< 0.0001
	HD	9 (3.5%)	3 (2.0%)	6 (5.6%)	
	NHL	21 (8.2%)	19 (12.7%)	2 (1.9%)	
	Brain tumor	85 (33.1%)	36 (24.0%)	49 (45.8%)	
	Nasopharyngeal ca	3 (1.2%)	0 (0%)	3 (2.8%)	
	Other	94 (36.6%)	62 (41.3%)	32 (29.9%)	
Gender	Male	147 (57.2%)	85 (56.7%)	62 (57.9%)	0.8384
	Female	110 (42.8%)	65 (43.3%)	45 (42.1%)	
Age at diagnosis, yrs	Mean ± SD	10.7 ± 5.3	10.9 ± 5.4	10.5 ± 5.2	0.5947
	Median (Min-max)	11.8 (0.1–19.8)	12.0 (0.4–19.8)	11.2 (0.1–19.3)	
Time from completion of therapy	Median (Min-max)	9.6 (5.0–19.5)	9.7 (5.0–19.5)	9.3 (5.0–16.7)	0.2324
Time from diagnosis, yrs	Median (Min-max)	11.2 (5.4–29.5)	11.2 (5.4–29.5)	11.0 (5.5–19.8)	0.8876
Chemotherapy only	No	135 (52.5%)	61 (40.7%)	74 (69.2%)	< 0.0001
	Yes	122 (47.5%)	89 (59.3%)	33 (30.8%)	
SCT	No	214 (83.3%)	133 (88.7%)	81 (75.7%)	0.0069
	Yes	43 (16.7%)	17 (11.3%)	26 (24.3%)	
Irradiation group 1	No	144 (56%)	103 (68.7%)	41 (38.3%)	<.0001
	Yes	113 (44%)	47 (31.3%)	66 (61.7%)	
CSI	No	210 (81.7%)	135 (90%)	75 (70.1%)	< 0.0001
	Yes	47 (18.3%)	15 (10%)	32 (29.9%)	
Cranial and head	No	221 (86%)	129 (86%)	92 (86%)	0.9966
	Yes	36 (14%)	21 (14%)	15 (14%)	
Spinal	No	252 (98.1%)	147 (98.0%)	105 (98.1%)	0.9403
	Yes	5 (1.9%)	3 (2.0%)	2 (1.9%)	
TBI	No	248 (96.5%)	148 (98.7%)	100 (93.5%)	0.0365
	Yes	9 (3.5%)	2 (1.3%)	7 (6.5%)	
Neck, nasopharynx	No	245 (95.3%)	146 (97.3%)	99 (92.5%)	0.0716
	Yes	12 (4.7%)	4 (2.7%)	8 (7.5%)	
Irradiation group 2 (Abdomen/pelvis/limbs)	No	235 (91.4%)	136 (90.7%)	99 (92.5%)	0.6572
	Yes	22 (8.6%)	14 (9.3%)	8 (7.5%)	

*yrs* Years, *SCT* Stem cell transplantation, *CSI* Craniospinal irradiation, *TBI* Total body irradiation, *MDS* Myelodysplastic syndrome, *HD* Hodgkin lymphoma, *NHL* Non-Hodgkin lymphoma

### Prevalence of the different thyroid abnormalities and clinical course

Of 257 subjects, transient or persistent thyroid abnormalities were identified in 107 (41.6%). Sixty-five out of 257 (25.3%) had subclinical hypothyroidism, and 16 (6.2%) developed central hypothyroidism. Five CCSs (1.9%) had primary overt hypothyroidism. 5 (1.9%) and 6 (2.3%) CCSs were diagnosed with autoimmune thyroiditis and thyroid cancer, respectively. Of 5 CCSs with autoimmune thyroiditis, 2 patients received SCT with TBI as a conditioning regimen. Of 6 CCSs with thyroid cancer, 4 were exposed to radiation therapy (either nasopharynx, lung, mediastinum or TBI). The initial thyroid hormone test results were not available in 10 CCSs with brain tumors because they were brought to the National Cancer Center with thyroxine medication from the diagnosis at another hospital. Nevertheless, the 10 CCSs were regarded as having persistent thyroid abnormalities due to necessity of thyroxine medication.

Among the different diagnostic groups, all nasopharyngeal cancer (*n* = 3) patients had thyroid abnormalities. Two with nasopharyngeal cancer had subclinical hypothyroidism, and the other patient had primary overt hypothyroidism, with TSH levels over 10 mU/L in all 3 patients. Thyroid abnormalities were found in 66.7% (6 out of 9) and 57.6% (49 out of 85) of the HD and brain tumor patients, respectively (Table 1). The chi-square test indicated a significantly different thyroid abnormality rate between different diagnoses (Table 1). The irradiation group 1 had more CCSs (58.4%) with thyroid abnormalities compared to the remaining CCSs (28.5%) (*P* < 0.0001) (Table 1). CCSs with SCT had an increased prevalence of thyroid abnormalities (60.5%) compared to the other CCSs (37.9%) (*P* = 0.0069) (Table 1).

The time to identify thyroid abnormalities from diagnosis of primary cancer was a median of 3.3 (0–14.8) years (Table 2). The cumulative event rate (thyroid abnormalities) was 21.2, 30.3, and 43.6% at 3 years, 5 years, and 10 years, respectively, after diagnosis of primary cancer. At the end of cancer therapy, 34 of 257 CCSs had developed thyroid abnormalities. Seventy-three CCSs exhibited thyroid abnormality during follow-up period after cancer treatment. The time to detect thyroid abnormalities from the end of treatment of cancer was a median of 1.3 (0–14.4) years in 107 subjects with thyroid abnormalities. Forty-five (42%) of 107 subjects with thyroid abnormalities had transient thyroid abnormalities. Forty (88.9%) of 45 transient thyroid abnormality were subclinical hypothyroidism. At the last follow-up, 62 CCSs of 257 CCSs (24.1%) had thyroid abnormalities. Forty-six of 58 (79.3%) non-brain tumor CCSs had subclinical hypothyroidism, showing transient thyroid abnormalities were more common in non-brain tumor CCSs compared to CCSs with brain tumors. The median follow-up time after completing cancer therapy was 9.6 years (5–16.7) and 9 years (5–16.6) in transient and persistent thyroid abnormalities, respectively. Thirty-five (70%) out of 50 CCSs with subclinical hypothyroidism (4.5 mU/L ≤ TSH < 10 mU/L) showed normalized thyroid hormone tests at the last follow-up. Ten (66.7%) out of 15 with subclinical hypothyroidism (TSH ≥ 10 mU/L) had persistent subclinical hypothyroidism (Table 2). Twenty-one CCSs with primary overt hypothyroidism or central hypothyroidism were categorized into persistent thyroid abnormalities because all 21 have been on thyroxine medication at the last follow-up.

**Table 2:** Clinical course of thyroid abnormalities

Initial presentation	N	Median time from diagnosis of cancer (years)	Thyroid abnormalities (transient case)			
			Brain tumors	No Brain tumors	Irradiation group 1	Chemo only or irradiation group 2
Total	107	3.3(0–14.8)	49(11)	58(34)	73(24)	34(21)
Normal freeT4, TSH 4.5–10 mU/L	50	5.5(0–14.8)	13(9)	37(26)	28(18)	22(17)
Normal freeT4, TSH ≥ 10 mU/L	15	2.1(0–5.0)	6(1)	9(4)	13(4)	2(1)
Decreased freeT4, normal TSH	16	0.5(0–14.2)	16(0)	0(0)	13(0)	3(0)
Decreased freeT4, TSH 4.5–10 mU/L	2	0/0.1	2(0)	0(0)	2(0)	0(0)
Decreased freeT4, TSH ≥ 10 mU/L	3	2.2(0–3.4)	1(0)	2(0)	1(0)	2(0)
Autoimmune thyroiditis	5	1.0(0.7–4.7)	1(1)	4(2)	2(1)	3(2)
Secondary thyroid cancer	6	7.5(1.0–10.5)	0(0)	6(2)	4(1)	2(1)
Unknown	10	0	10(0)	0(0)	10(0)	0(0)

*FreeT4* Free thyroxine, *TSH* Thyroid stimulating hormone; The irradiation fileds of group 1 included head and neck, craniospinal irradiation, total body, cervical spine, nasopharynx, and mediastinum. The remaining irradiation fields (abdomen, pelvis, and limbs) were regarded as irradiation group 2

### Treatment factors related thyroid abnormalities

**Fig. 1:**
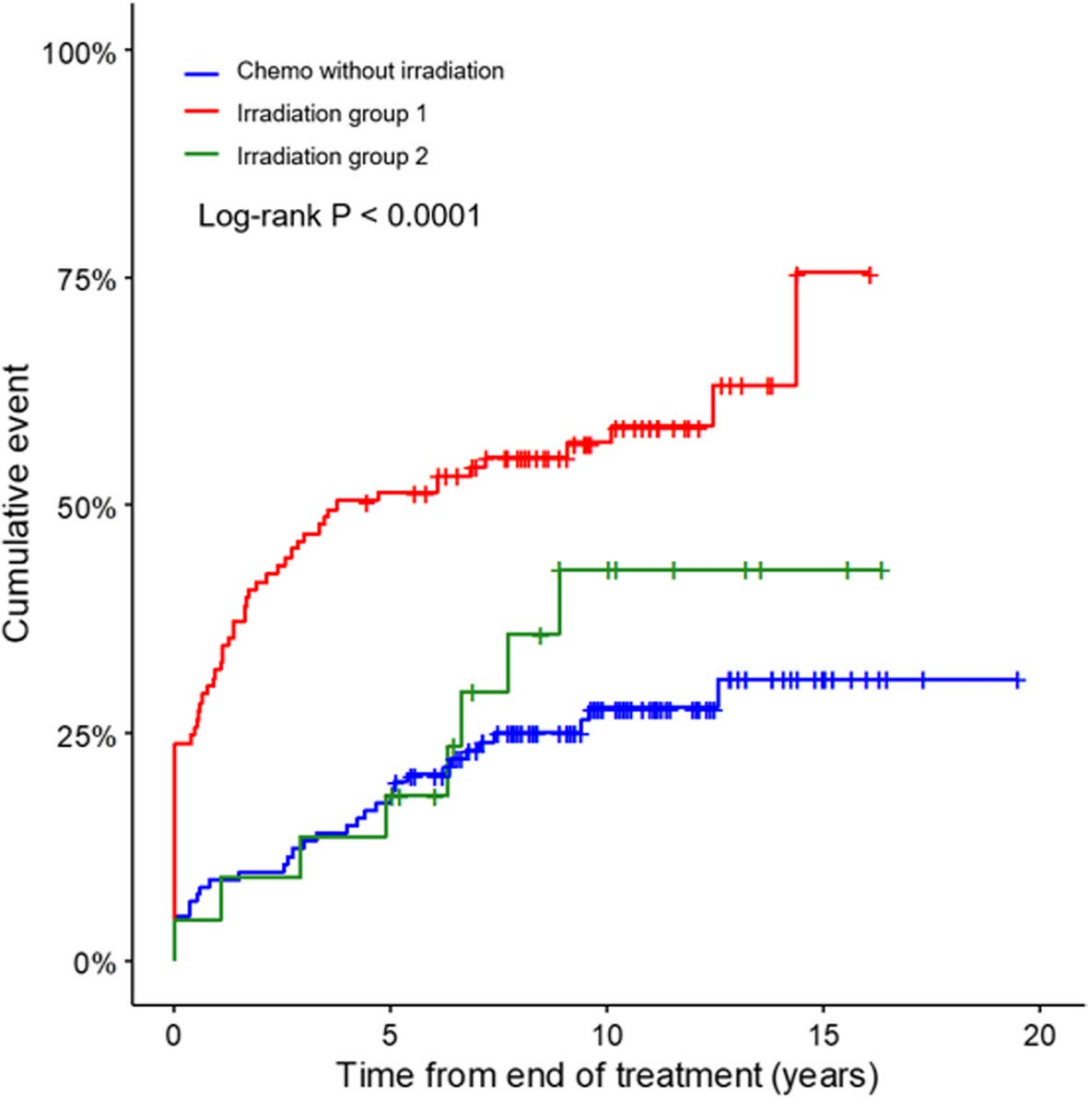
Cumulative incidence in different treatment subgroups of childhood cancer survivors. The log-rank test showed a significant difference between different treatment groups. The irradiation group 1 had the highest event rate compared to the other treatment groups. Irradiation field of group 1 were head and neck, craniospinal irradiation, total body irradiation, cervical spine, nasopharynx, and mediastinum. The remaining irradiation fields (abdomen, pelvis, and limbs) were regarded as irradiation group 2

**Fig. 2:**
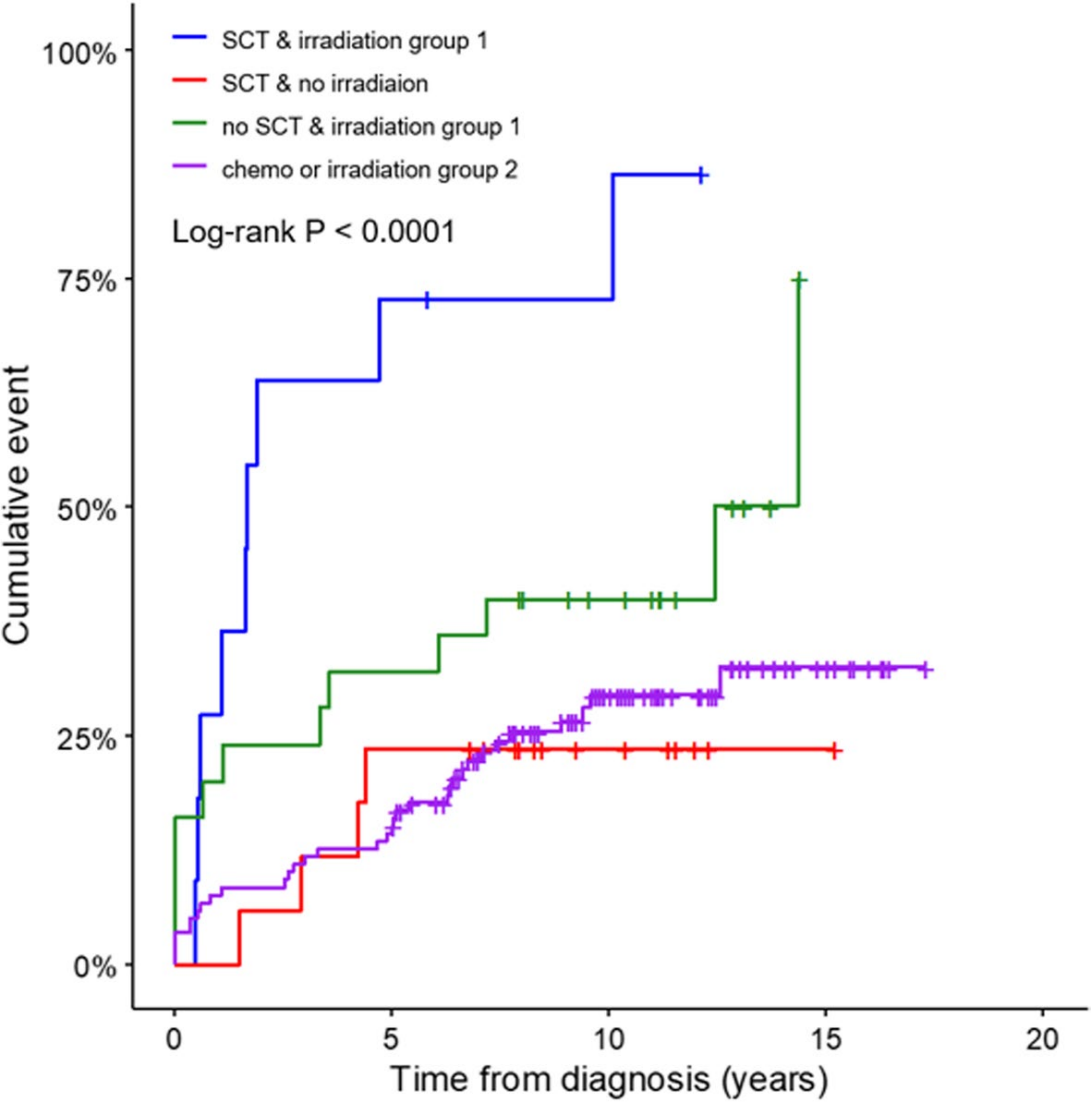
Cumulative incidence in different treatment subgroups of childhood cancer survivors (CCSs). The highest thyroid abnormality rate was observed in CCSs who received stem cell transplantation (SCT) accompanied by irradiation directly or near hypothalamic-pituitary-thyroid axis

The irradiation group 1 had the highest event rate compared to the chemo only or irradiation group 2 (*P* < 0.0001) (Fig. 1). CCSs who received TBI and neck/nasopharynx had thyroid abnormalities in 7 of 9 subjects and 8 of 12 subjects, respectively. However, among different radiation fields such as CSI, head, TBI, and neck/cervical/nasopharynx/spinal areas, there was no significant thyroid abnormalities rate (*P* = 0.0592). Among 65 CCSs with subclinical hypothyroidism, only 2 subjects had TSHs level above 10 mU/L in 24 CCSs who did not received radiotherapy, while 13 of 41 irradiated CCSs had TSH levels over 10 mU/L (*P* = 0.0309). Twenty-two (53.7%) of 41 irradiated subjects had normalized TSH levels, whereas 18 (75%) of the remained 24 non-irradiated subjects had normalized TSH levels at the last follow-up (Table 2). Of 107 subjects with thyroid abnormalities, transient cases were more prevalent in chemo only group than the other enrolled CCSs (*P* = 0.0025), and persistent cases were more common in irradiation group 1 than the rest CCSs (*P* = 0.0004). In the analysis to observe the effect of SCT, there was a statistically significant difference among the 4 different groups (Fig. 2) (*P* < 0.0001). The highest event rate was found in CCSs treated with both SCT and radiation therapy near HPT axis or TBI. Multiple comparisons using Bonferroni method were shown in suppl table. Two patients with sarcoma had been on tyrosine kinase inhibitor, and 1 patient with brain tumor used to take bevacizumab. Among those 3 subjects, 2 had transient thyroid dysfunction and 1 had persistent thyroid dysfunction.

## Discussion

Thyroid function is essential for growth and development in children and adolescents. In addition, T3 and TSH hormone concentrations are also associated with impaired emotional and physical health aspects related to the quality of life [14]. Thus, early recognition and treatment of thyroid disease are required in CCSs. This study showed that thyroid abnormalities were frequently observed and the time interval between cancer diagnosis and the identification of thyroid abnormalities varied, necessitating regular monitoring for thyroid function. Because of variability of thyroid disorders in CCSs, investigating risk factors for thyroid abnormalities based on diagnosis and treatment modalities is helpful for follow-up care of CCSs.

Among the different diagnostic groups, thyroid abnormalities were frequent in the brain tumor or HD or nasopharyngeal cancer groups. Brain tumors such as germ cell tumors, Langerhans histiocytosis, craniopharyngioma and pituitary adenoma involving the hypothalamus or pituitary gland area were accompanied by thyroid hormone deficiencies at diagnosis, leading to development of central hypothyroidism and the higher prevalence of persistent thyroid abnormalities than in other CCSs. In addition, transient or persistent subclinical hypothyroidism was also commonly found in brain tumor CCSs as well as in the other CCSs. Among CCSs with subclinical hypothyroidism, over half had normalized thyroid function at the last follow-up. Irradiation near HPT axis area or TBI were a predicting factor of developing persistent subclinical hypothyroidism.

Radiation therapy has been considered a risk factor for hypothyroidism, thyroid nodule, and thyroid cancer [1]. Nevertheless, radiotherapy is an inevitable treatment modality for some childhood cancers. Our study also showed that the CCSs irradiated near HPT axis (irradiation group 1) had more thyroid abnormalities than the chemotherapy only or abdomen/pelvis/limbs irradiation groups (irradiation group 2).

CSI has been used for patients with brain tumors, which tend to spread via the cerebrospinal fluid. CSI poses a risk of thyroid disease due to the treatment field, which includes the hypothalamic-pituitary and cervical areas, resulting in the development of primary or secondary or mixed hypothyroidism. Primary hypothyroidism including transient hypothyroidism was reported to be found in 37.5–73% of the patients with CSI [9, 15, 16]. Radiation exposure over 25 Gy to the thyroid gland could lead to primary hypothyroidism due to direct tissue injury. Secondary hypothyroidism develops by radiation exposure higher than 30 Gy to the hypothalamus-pituitary axis [17]. The prevalence of secondary hypothyroidism after cranial radiation was reported to be 7.5–59% in previous studies [16, 18–20]. A previous study suggested that partial central hypothyroidism could remain undetected by a one-time thyroid hormone test without a provocation test in out-patient clinics, indicating that central hypothyroidism could be much more common [21]. Because a scattered dose of radiation to the thyroid gland after CSI cannot be precisely measured, it is difficult to compare the incidence rate of thyroid disease according to the CSI dosage. A few previous studies found that the CSI dose was not a significant predictor of hypothyroidism [9, 15]. In addition, in low-dose radiation exposure, thyroid cancer risk rather than hypothyroidism was increased in a linear dose-responsive manner, necessitating continuous monitoring regardless of the irradiation dose [22].

Our study showed a higher prevalence of thyroid abnormalities when high dose chemotherapy (SCT) was accompanied by irradiation directly or near HPT axis area compared to the SCT without irradiation and the irradiation near HPT axis area without SCT. Although some chemotherapeutic agents are known to affect thyroid cells, the impact of chemotherapeutic agents on the thyroid gland remains inconclusive [23, 24]. Thus, it is not clear if there were some combined or synergistic effects on thyroid cells of chemotherapy and radiation therapy, and the mechanism needs to be clarified. As for development of autoimmune thyroiditis after SCT, several studies have reported autoimmune thyroiditis after SCT by the transfer of abnormal B- or T-cells clones from the donor to the host [25, 26]. However, it is not certain whether thyroid auto-Abs were present before the cancer diagnoses in this study. In case thyroid auto-Abs are present before a cancer diagnosis, chemotherapy or irradiation could be a risk of inducing autoimmune thyroiditis [27].

This study has limitations. First, follow-up periods were various. Thus, some CCSs could present thyroid abnormalities later, and thyroid abnormalities regarded as persistent at the last follow-up could turn out to be transient in the future. Secondly, TSH measurement frequency was highly different depending on individuals, which could affect detection time of thyroid disorder. TSH measurement time was also diverse, which could slightly make an impact on TSH levels. Thirdly, thyroid disorders could be under or overestimated depending on surveillance modalities. In the present study, thyroid auto Abs measurement or ultrasound examination were not conducted in all subjects, which could impact on prevalence of thyroid nodules, cancer or autoimmune thyroid disease.

In our study, subclinical hypothyroidism was common in CCSs, which is consistent finding with the previous report [8]. Thyroxine medication is generally recommended when the TSH concentration is over 10 mU/L in subclinical hypothyroidism. When the TSH levels were under 10 mU/L, subclinical hypothyroidism was transient in substantial portion of the cases, suggesting that there is no need for medical treatment. Nevertheless, thyroxine medication in patients with persistent subclinical hypothyroidism with TSH levels lower than 10 mU/L should be investigated from the perspective of cardiovascular disease risk, thyroid cancer risk, and other health conditions related to the quality of life. According to the Children’s Oncology Group guidelines, a thyroid physical examination and free T4 and TSH testing are recommended yearly for CCSs with irradiation and more frequent screening should be considered during periods of rapid growth. Furthermore, thyroid abnormalities are sometimes detected during cancer treatment. Additional therapies to chemotherapy or radiotherapy such as TKIs and immune check point inhibitors could heighten the risk of thyroid abnormalities, requiring regular check-ups for thyroid function during cancer therapy based on a risk for thyroid abnormalities. In addition, it is controversial whether periodic ultrasound-based screening is necessary to detect thyroid nodule or cancer in the early stages [28–30].

## Conclusions

Thyroid abnormalities were prevalent and subclinical hypothyroidism was most common in CCSs. CCSs with irradiation directly or near HPT axis were at risk for persistent thyroid dysfunction. The elapsed time for detecting thyroid abnormalities varied, necessitating continuous surveillance.

## Supplementary Information

**Additional file 1: Supplement table 1.** Multiple comparison tests using Bonferroni method.

## Data Availability

The datasets used during the current study available from the corresponding author on reasonable request.

## Abbreviations

CCSs: Childhood cancer survivors
HPT: Hypothalamus-pituitary-thyroid
TBI: Total body irradiation
PNET: Peripheral neuroectodermal tumor
TKIs: Tyrosine kinase inhibitors
SCT: Stem cell transplantation
MDS: Myelodysplastic syndrome
HD: Hodgkin lymphoma
NHL: Non-Hodgkin lymphoma
CSI: Craniospinal irradiation
ATRTs: Atypical teratoid/rhabdoid tumors
fT4: Free thyroxine
TSH: Thyroid-stimulating hormone
TPOAbs: Antithyroid peroxidase antibodies
TgAbs: Anti-thyroglobulin antibodies
