# Supplementary material for: Characteristics and clinical course of thyroid abnormalities arisen in long term survivors of childhood cancer
Source: BMC Pediatr. 2023 Mar 18;23:124. doi: 10.1186/s12887-023-03900-x (PMC10024379; doi:10.1186/s12887-023-03900-x)
Supplement: Supplementary file 1 — Additional file 1: Supplement table 1. Multiple comparison tests using Bonferroni method. [file 12887_2023_3900_MOESM1_ESM.docx]

Supplement table 1. Multiple comparison tests using Bonferroni method.

| Comparison between different treatment modalities | | *P* value |
| --- | --- | --- |
| Chemo without irradiation | Irradiation group 1 | <0.0001 |
| Chemo without irradiation | Irradiation group 2 | 0.0022 |
| Irradiation group 1 | Irradiation group 2 | <0.0001 |
| SCT and irradiation group 1 | SCT and no irradiation | 0.0093 |
| SCT and irradiation group 1 | no SCT and irradiation group 1 | 1 |
| SCT and irradiation group 1 | chemo or irradiation group 2 | 0.0005 |
| SCT and no irradiation | no SCT and irradiation group 1 | 0.6831 |
| SCT and no irradiation | chemo or irradiation group 2 | 0.8927 |
| no SCT and irradiation group 1 | chemo or irradiation group 2 | 0.1005 |

SCT, stem cell transplantation; Irradiation field of group 1 were head and neck, craniospinal irradiation, total body irradiation, cervical spine, nasopharynx, and mediastinum. The remaining irradiation fields (abdomen, pelvis, and limbs) were regarded as irradiation group 2.
